# Different choline supplement metabolism in adults using deuterium labelling

**DOI:** 10.1007/s00394-023-03121-z

**Published:** 2023-02-25

**Authors:** Katrin A. Böckmann, Axel R. Franz, Anna Shunova, Michaela Minarski, Cornelia Wiechers, Christian F. Poets, Wolfgang Bernhard

**Affiliations:** 1 Department of Neonatology, Faculty of Medicine, Eberhard Karls University, Calwer Straße 7, 72076 Tübingen, Germany; 2 Center for Pediatric Clinical Studies, Eberhard Karls University, Tübingen, Germany

**Keywords:** D9-betaine, D9-choline, D9-phosphatidylcholine remodelling, D9-phosphocholine, D9-glycerophosphocholine, Trimethylamine oxide

## Abstract

**Background:**

Choline deficiency leads to pathologies particularly of the liver, brain and lung. Adequate supply is important for preterm infants and patients with cystic fibrosis. We analysed the assimilation of four different enterally administered deuterium-labelled (D9-) choline supplements in adults.

**Methods:**

Prospective randomised cross-over study (11/2020–1/2022) in six healthy men, receiving four single doses of 2.7 mg/kg D9-choline equivalent each in the form of D9-choline chloride, D9-phosphorylcholine, D9-alpha-glycerophosphocholine (D9-GPC) or D9-1-palmitoyl-2-oleoyl-glycero-3-phosphoryl-choline (D9-POPC), in randomised order 6 weeks apart. Plasma was obtained at baseline (*t* = − 0.1 h) and at 0.5 h to 7d after intake. Concentrations of D9-choline and its D9-labelled metabolites were analysed by tandem mass spectrometry. Results are shown as median and interquartile range.

**Results:**

Maximum D9-choline and D9-betaine concentrations were reached latest after D9-POPC administration versus other components. D9-POPC and D9-phosphorylcholine resulted in lower D9-trimethylamine (D9-TMAO) formation. The AUCs (0-7d) of plasma D9-PC concentration showed highest values after administration of D9-POPC. D9-POPC appeared in plasma after fatty acid remodelling, predominantly as D9-1-palmitoyl-2-linoleyl-PC (D9-PLPC), confirming cleavage to 1-palmitoyl-lyso-D9-PC and re-acylation with linoleic acid as the most prominent alimentary unsaturated fatty acid.

**Conclusion:**

There was a delayed increase in plasma D9-choline and D9-betaine after D9-POPC administration, with no differences in AUC over time. D9-POPC resulted in a higher AUC of D9-PC and virtually absent D9-TMAO levels. D9-POPC is remodelled according to enterocytic fatty acid availability. D9-POPC seems best suited as choline supplement to increase plasma PC concentrations, with PC as a carrier of choline and targeted fatty acid supply as required by organs. This study was registered at Deutsches Register Klinischer Studien (DRKS) (German Register for Clinical Studies), DRKS00020498, 22.01.2020.

**Study registration:**

This study was registered at Deutsches Register Klinischer Studien (DRKS) (German Register for Clinical Studies), DRKS00020498.

**Supplementary Information:**

The online version contains supplementary material available at 10.1007/s00394-023-03121-z.

## Introduction

Nutritional supply of choline as an essential nutrient is often not sufficient [1–3], but needed for cell membrane formation of all tissues and many secretions (bile, lipoproteins, surfactant). It is also required for the synthesis of the neurotransmitter acetylcholine, fundamental to foetal synaptogenesis, development and function of the brain as well as to leucocyte function [4–6]. Via phosphatidylcholine (PC), choline is essential for sphingomyelin synthesis from (pro-apoptotic) ceramides, and via its downstream metabolite, betaine, it is important as a methyl donor used for methionine regeneration from homocysteine and to kidney function [7]. In utero, the foetus is supplied with free choline via active placental transport, whereas breast milk predominantly contains phosphorylcholine and alpha-glycerophosphorylcholine (GPC). After weaning, PC is the predominant choline carrier in regular food [8].

In preterm infants, plasma levels of choline rapidly fall by 50% after birth, leading to an unphysiological decrease during a time of exponential growth and choline requirements [1]. Patients with cystic fibrosis (CF) also have a risk of severe choline deficiency, caused by exocrine pancreas insufficiency and deficiency of phospholipase A2 activity being required to assimilate PC of food and recycle PC of bile [9].

The liver’s choline homeostasis is important to secrete bile PC for triglyceride emulsification and to synthesise lipoprotein PC for the assembly and secretion of very low-density lipoproteins (VLDL). Notably, choline can be mobilised from the lung as high-density lipoprotein PC, which may become ‘choline-exhausted’ as it comprises only ¼ of the hepatic choline pool, to compensate for the liver’s choline deficiency [10, 11]. In line with this, both liver and lung function in CF patients correlated with choline status [3, 10, 12] and this observation may also be relevant for preterm infants [1].

Choline requirement is not covered by endogenous synthesis. Choline was, therefore, defined as an essential nutrient by the US-Food and Drug Administration (FDA) and National Academy of Medicine of the USA (NAM) in 1998 and European Food Safety Authority (EFSA) by 2016. NAM defined the adequate intake (AI) for male adults as 550 mg/d (= ~ 7–8 mg/kg/d) [13]. Nevertheless, choline requirements vary with sex, age, genetic polymorphisms, folic acid intake and lifestyle [14–16]. Moreover, supplementation of choline may have side effects differing amongst the various choline components. Choline may be partly degraded to trimethylamine (TMA) by small intestinal bacteria and hence may not be fully available to the host. TMA, transported to the liver via the portal vein, is oxidised to trimethylamine oxide (TMAO), which may be a cardiovascular risk factor. Recent data indicate differences in TMAO formation following enteral administration of different choline components [17, 18].

In contrast to choline salts (like choline chloride) as common components in choline supplements, nutritional choline is physiologically provided as organic esters, with phosphorylcholine and GPC dominating in breast milk and PC dominating in regular food after weaning. We aimed to analyse differences in the metabolism and kinetics of these different choline supplements to define their differences in assimilation and effects on plasma concentration of choline and its metabolites and on TMAO formation. Aim was finding an effective and safe supplement resulting in low levels of TMAO in plasma after administration. This will be important for patient groups with typically low levels of choline like preterm infants or patients with cystic fibrosis. Beyond this, we found that PC as a choline supplement, other than water-soluble compounds, substantially affected the metabolism of polyunsaturated fatty acids (LC-PUFA), therefore possibly being a tool in concert with LC-PUFA supplementation. We used D9-choline chloride, D9-phosphorylcholine, D9-GPC and D9-POPC as stable-isotope-labelled analogues of the respective natural choline supplements.

## Methods

This is a randomised cross-over study, carried out at the Department of Neonatology, Tübingen University Hospital, Germany. Six healthy adult men were recruited and studied between November 2020 and January 2022. The Institutional Review Board (project number 322/2019BO1) approved the protocol, and written informed consent was obtained prior to enrolment.

### Inclusion criteria

Test persons had to be male and at least 18 years old.

Exclusion criteria were alcohol abuse, acute illness, chronic diseases (like diabetes, metabolic syndrome, thyroid diseases, pancreas insufficiency), intake of choline containing nutritional supplements or missing consent.

### Supplements

The deuterium-labelled (D9) analogues choline-d9 chloride [N,N,N-trimethyl-d9] (D9-choline chloride), D9-phosphorylcholine chloride, calcium salt (D9-phosphorylcholine), D9-alpha-glycerophosphorylcholine (D9-GPC) and D9-1-palmitoyl-2-oleoyl-glycero-3-phosphorylcholine (D9-POPC) were from EQ Laboratories GmbH (Augsburg, Germany). Pure substances were aliquoted into screw capped opaque glass vials by Rainfarn-Apotheke (RAINFARN Gesundheit, Munich, Germany). Substances were delivered as dry substance for oral application in suitable quantity for a single administration of 350 mg D9-choline chloride, 620 mg D9-phosphorylcholine, 620 mg D9-GPC and 1.8 g D9-POPC to achieve a single dose of 2.7 mg/kg D9-choline equivalent.

### Study schedule

Directly prior to use, substances were dissolved/emulsified in 10 ml sterile water and topped up with 250 ml apple spritzer. Test subjects had to ingest in a randomised sequence at least 6 weeks (w) apart D9-choline metabolite for wash out.

- D9-choline chloride (3.6 mg/kg),
- D9-phosphorylcholine chloride (6.4 mg/kg),
- D9-alpha GPC (6.4 mg/kg) or
- D9-POPC (18.6 mg/kg)

Experiments started in the morning after overnight fasting, and the supplement was served in 250 mL apple spritzer together with a butter pretzel (102 g) containing 16.9 mg total choline and 10.3 mg betaine as well as 12 g fat. Detailed composition of the study meal fatty acids is shown in Table s1 of the online supplement.

Blood (2.7 ml EDTA) was taken before intake (− 0.1 h) and at 0.5 h, 1 h, 1.5 h, 2 h, 3 h, 4 h, 5 h, 6 h, 9 h, 24 h, 33 h and 48 h, 3d, 4d and 7d after D9-choline intake. Test persons fasted until the 6 h-blood sample was collected. Collected blood was immediately centrifuged at 1000x*g* at room temperature for 10 min, plasma was separated and stored at -80 °C until analysis.

### Chemical analysis

The analyses of deuterium-labelled choline and its metabolites were performed with electrospray ionisation tandem mass spectrometry (ESI–MS/MS) as previously described [3, 19, 20]. In brief, water-soluble components (like D9-choline, -betaine, -phosphorylcholine and -GPC) were separated on a HILIC Plus^®^ column (Agilent Technologies, Böblingen, Germany) at 40 °C with an acetonitrile: water: ammonium formate gradient, whereas D9-PC, D3-PC, -lyso-PC and -SPH were separated from other phospholipids on a Polaris 3 Si-A column^®^ (Agilent Technologies) at 40 °C with chloroform: methanol:300 mM ammonium acetate (60:38:2,vol/vol) as the mobile phase, with flow rates of 400µL/min for native and D9-labelled compounds and 200µL/min for D3-PC to increase sensitivity. Detection was at positive ionisation in the SRM mode for the respective diagnostic fragments [20], These have mass/charge (m/z) ratios of + 184 for native PC, + 193 for D9-choline-labelled PC being synthesised by de novo synthesis (D9-PC) and + 187 for D3-labelled PC from sequential methylation of phosphatidylethanolamine (PE) via the PEMT pathway (D3-PC) [7]. D9-PC and D3-PC were quantified as the sums of all detected D9/D3-PC molecular species (for details see: [3, 7, 10]). For further sub-grouping of D9-PC, individual molecular species were then categorised by their content of an oleic acid [like D9-palmitoyl-oleoyl-PC and D9-stearoyl-oleoyl-PC] (D9-C18:1-PC), a linoleic acid [like D9-palmitoyl-linoleyl-PC and D9-stearoyl-linoleyl-PC] (D9-C18:2-PC), an arachidonic acid [like D9-palmitoyl-arachidonoyl-PC and D9-stearoyl-arachidonoyl-PC] (D9-C20:4-PC) or a docosahexaenoic acid [like D9-palmitoyl-docosahexaenoyl-PC and D9-stearoyl-docosahexaenoyl-PC] (D9-C22:6-PC) residue as described in detail before [20].

### Statistics

*D*ifferences between the plasma kinetics and metabolization of D9-labelled choline carriers in adults are unclear, as there are no comparative data on this in humans or animals [21]. Therefore, calculation of group sizes was explorative, assuming a normal distribution. In adults, plasma choline concentrations were 5.2 ± 1.6 µmol/L without and 13.7 ± 5.3 µmol/L with supplementation (2200 mg/d equivalent to 34 mg/kg/d at a mean body weight of 65 kg) [3]. Assuming normally distributed data with equal sample size, use of analysis of variance for unrelated samples (because no data on related samples was available at that time), and an *α* ≤ 0.05 with a power of 80%, it was estimated that at least six subjects must be studied to demonstrate clinically relevant differences in mean concentration increases and area under the curve (AUC) of plasma D9-choline between test substances.

AUC of D9-choline plasma concentrations was chosen as primary outcome. Secondary outcome parameters were AUCs and the maximum plasma concentrations of D9-choline metabolites (D9-betaine, D9-PC), time to reach the maximum plasma concentrations of D9-choline and its derivatives, and differences in concentrations over time of the D9-PC sub-groups resulting from D9-choline tracer assimilation and metabolism. JMP 14 (SAS Institute GmbH, Germany), and Excel 2010 (Microsoft Corporation, USA) were used for statistical and graphical analyses. AUCs were calculated using the trapezoidal rule. Normal distribution was tested with Shapiro–Wilk test. Because several parameters were not normally distributed, data are shown as median with 25th and 75th percentile and non-parametric Wilcoxon test were used. Correction for multiple testing was not applied, *p < *0.05 was regarded as significant.

## Results

Demographic data of the study subjects are shown in Table 1. Graphic presentation of demographic data in correlation with AUC of choline and betaine is shown in graph 1–4 of the online supplement.

**Table 1:** Demographic data of study participants

Number of participants	6
Age (years)	37.5 (29.75/56) [26–65]
Weight (kg)	72 (68/84) [57–93]
Height (cm)	184.5 (176.5/187) [175–190]
BMI (kg/m^2^)	21.9 (20.2–24.4) [18.2–27.2]

Data are shown as median, (interquartile range) and [full range]

*BMI* body mass index

### D9-choline and its water-soluble downstream metabolites

**Fig. 1:**
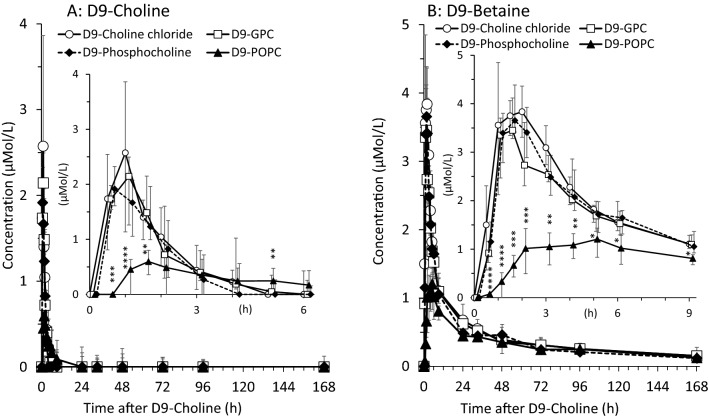
Plasma kinetics of D9-choline (**A**) and D9-betaine (**B**) in response to deuterium-labelled (D9) choline supplements. Inserts also show concentrations over time, focussing on a shorter time period (6, 9 h). Data are shown as medians and interquartile ranges. *D9* ninefold deuterium labelled at the trimethylammonium group of the choline moiety; *D9-GPC* D9-glycerophosphorylcholine; *h* hours; *D9-POPC* D9-1-palmitoyl-2-oleoyl-phosphatidylcholine; *p < 0.001 D9-choline chloride vs. other compounds; †*p < *0.01 D9-GPC vs. other compounds

Figure 1A demonstrates that, after single oral intake, plasma D9-choline reached maximum median concentrations of 2.36[1.54–2.65]µmol/L at *t*_max_ = 0.5–1 h, with no significant differences between hydrophilic compounds (D9-choline, D9-phosphocholine, D9-glycerophosphocholine; *p > *0.05). Notably, D9-phosphocholine had its maximum at 0.5 h, whereas D9-choline chloride and D9-GPC at 1 h. After 5 h and beyond, plasma D9-choline had returned to 0 µmol/L for all these compounds.

By contrast, ingestion of D9-POPC resulted in delayed (*t*_max_ = 1.5 h) and smaller (*c*_max_ = 0.60[0.43–0.75]µmol/L, *p < *0.01) peak concentrations of D9-choline, and also a more delayed return to zero concentrations beyond 9 h.

Similarly, the kinetics and maximum concentrations of D9-betaine were different in response to D9-POPC compared to water-soluble compounds (Fig. 1B). D9-choline chloride, D9-phosphocholine and D9-GPC rapidly increased, with a uniform *t*_max_ = 1.5 h and maximum D9-betaine values of 3.60[3.28–4.26]µmol/L, compared to 1.21[0.93–1.43]µmol/L found at 5 h after D9-POPC (*p < *0.001).

**Fig. 2:**
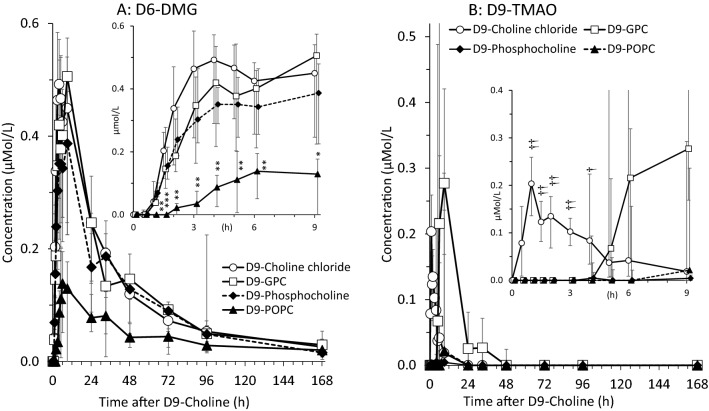
Plasma kinetics of D6-DMG (Panel A) and D9-TMAO (Panel B) in response to deuterium-labelled (D9-) choline supplements. Inserts also show concentrations over time, focussing on a shorter time period (9 h). Data are shown as medians and interquartile ranges. *D9* ninefold deuterium labelled at the trimethylammonium group of the choline moiety; *D6-DMG* D6-dimethylglycine; *D9-GPC* D9-glycerophosphorylcholine; *h* hours; *D9-POPC* D9-1-palmitoyl-2-oleoyl-phosphatidylcholine; *p < 0.001 vs. water-soluble

Finally, the concentrations of D6-dimethylglycine (D6-DMG), the demethylation product of D9-betaine used for (D3-)methionine synthesis from homocysteine, was lower after D9-POPC ingestion compared to the other deuterium-labelled compounds (Fig. 2A) (*p < *0.001). Here, the water-soluble compound slowly reached a plateau at 3–9 h of 0.40[0.22–0.53]µmol/L, compared to 0.10[0.05–0.16]µmol/L at 4–33 h for D9-POPC (*p < *0.001) (Fig. 2A).

### D9-TMAO

D9-TMAO was absent in response to both D9-POPC and D9-phosphocholine ingestion, but was detected after D9-choline chloride and D9-GPC, with a tendency to a later increase after D9-GPC (Fig. 2B).

### D9-choline-labelled phospholipids in response to different D9-choline compounds

**Fig. 3:**
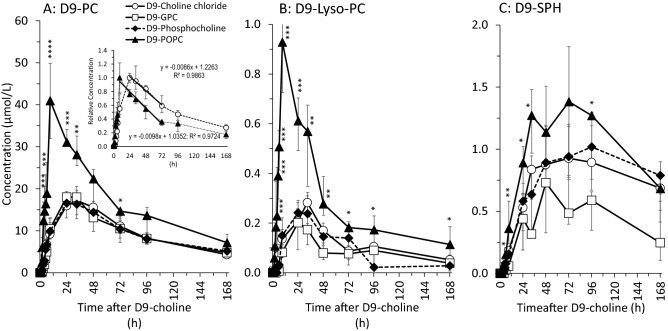
Plasma kinetics of the sum of D9-labelled phosphatidylcholine (D9-PC) (**A**), sum of D9-lyso-PC (**B**) and D9-sphingomyelin (D9-SPH) (**C**) in response to deuterium-labelled (D9-) choline supplements. Insert in A shows the relative decrease of plasma D9-PC during linear phase from max to 72 h. Data are shown as medians and interquartile ranges. *D9* ninefold deuterium labelled at the trimethylammonium group of the choline moiety; *D9-GPC* D9-glycerophosphocholine; *h*, hours; *D9-POPC* D9-1-palmitoyl-2-oleoyl-phosphatidylcholine. *p < 0.05, **p < 0.01, ***p < 0.001, ****p < 0.0001 D9-POPC vs. water-soluble D9-choline components

As demonstrated in Fig. 3A, water-soluble compounds exhibited a plateau of D9-PC at 24–33 h after their ingestion (17.6[16.3–18.7]µmol/L), with no significant differences amongst compounds. By contrast, after D9-POPC plasma D9-PC showed a more rapid and steeper increase that peaked at 9 h, with no plateau and maximum concentrations 2.3-fold higher (41.0[31.9–50.0]µmol/L; *p < *0.001) than after water-soluble compounds. Similar kinetics were seen for plasma D9-lyso-PC in response to D9-POPC compared to D9-choline chloride, D9-phosphocholine and D9-GPC (Fig. 3B), whereas plasma D9-SPH showed a delayed increase, with higher values after D9-POPC as well (Fig. 3C). Notably, whilst D9-POPC exhibited a 2.3-fold higher peak concentration of plasma D9-PC, the decrease of plasma D9-PC followed a similar kinetic as seen after water-soluble D9-choline compounds (Fig. 3A, insert), reaching comparable concentrations at 72 h. Consequently, after 72 h, D9-PC had decreased by 26.3[25.9–28.5]µmol/L after D9-POPC, but only by 7.2[4.6–8.92]µmol/L after water-soluble D9-choline supplements (*p < *0.01).

### Differential D9-PC metabolism in response to D9-POPC compared to water-soluble D9-choline supplements

**Fig. 4:**
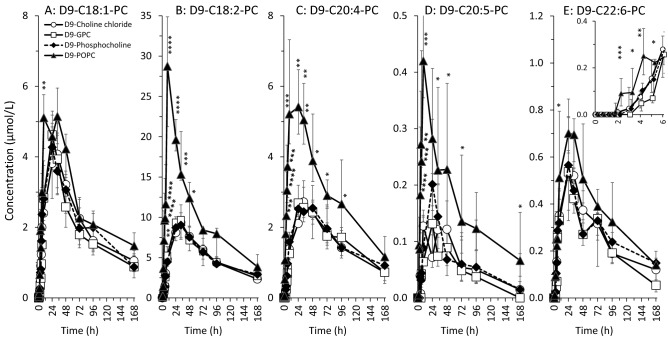
Plasma concentrations of D9-PC sub-groups in response to deuterium-labelled (D9-) choline supplements. D9-PC molecular species were sub-grouped into those containing an oleic (D9-C18:1-PC) (**A**), linoleic (D9-C18:2-PC) (**B**), arachidonic (D9-C20:4-PC) (**C**), eicosapentaenoic (D9-C20:5-PC) (**D**) or docosahexaenoic (D9-C22:6-PC) (**E**) acyl residue (for details see Materials and Methods). Data are shown as medians and interquartile ranges. *D9* ninefold deuterium labelled at the trimethylammonium group of the choline moiety; *D9-GPC* D9-glycerophosphorylcholine; *h* hours; *D9-POPC* D9-1-palmitoyl-2-oleoyl-phosphatidylcholine. *p < 0.05, **p < 0.01, ***p < 0.001, ****p < 0.0001 D9-POPC vs. water-soluble D9-choline components

**Fig. 5:**
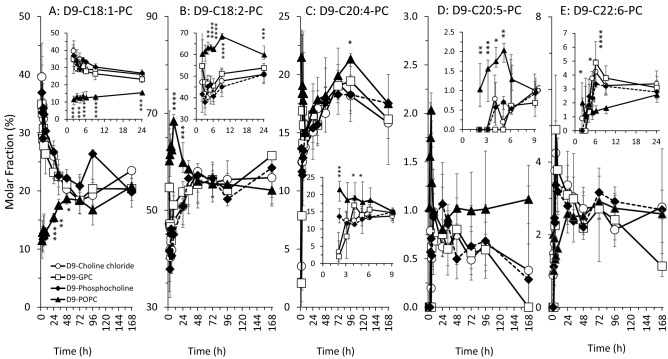
Molar fractions of D9-PC sub-groups in plasma in response to deuterium-labelled (D9-) choline supplements. D9-PC molecular species were sub-grouped into those containing an oleic (D9-C18:1-PC) (**A**), linoleic (D9-C18:2-PC) (**B**), arachidonic (D9-C20:4-PC) (**C**), eicosapentaenoic (D9-C20:5-PC (**D**) or docosahexaenoic (D9-C22:6-PC) (**E**) acyl residue (for details see Materials and Methods). Data are shown as medians and interquartile ranges. *D9* ninefold deuterium labelled at the trimethylammonium group of the choline moiety; *D9-GPC* D9-glycerophosphorylcholine; *h* hours; *D9-POPC* D9-1-palmitoyl-2-oleoyl-phosphatidylcholine. *p < 0.05, **p < 0.01, ***p < 0.001, ****p < 0.0001 D9-POPC vs. water-soluble D9-choline components

To differentially address the metabolism of fatty acids of PC in response to supplements, D9-PC was differentiated into sub-groups containing different unsaturated fatty acid residues [22], i.e. omega-9 monounsaturated oleic acid [C18:1] (to which D9-POPC belongs), the omega-6 compounds linoleic acid [C18:2] and arachidonic acid [C20:4], or the omega-3 compounds eicosapentaenoic acid [C20:5] and docosahexaenoic acid [C22:6]). Concentrations and molar fractions of these D9-PC sub-groups are shown in Figs. 4 and 5, respectively, generally representing the time course of total D9-PC after supplement ingestion.

Concentration changes of D9-C18:1-PC were virtually identical for all D9-choline supplements (Fig. 4A). However, its molar fraction was initially low and then increased in response to D9-POPC, whereas after water-soluble D9-choline supplements, it was initially high and then decreased, reaching equilibrium after 48 h (Fig. 5A). By contrast, both concentration (Fig. 4B) and fraction (Fig. 5B) of D9-C18:2-PC initially increased  ~ 2.5-fold after D9-POPC compared to water-soluble components, whilst equilibrium was achieved after 48 h as well. This similarly applied to D9-C20:4-PC and D9-C20:5-PC (Fig. 4C + D, Fig. 5C + D), however, with faster achievement of equilibrium concentrations. For D9-C22:6-PC, there were initially (2–6 h) higher concentrations after D9-POPC compared to the other supplements (Fig. 4E). Nevertheless, its molar fraction amongst PC sub-groups was initially lowest in response to D9-POPC due to higher increases of the other compounds (Fig. 5E).

### Areas under the curve of D9-choline and its metabolites

Areas under the curve were calculated for D9-choline and its deuterium-labelled metabolites as shown in Table 2. There were no significant differences between water-soluble D9-choline components, whereas AUC was significantly different for most plasma parameters in response to D9-POPC. After D9-POPC, AUC was lower for plasma D9-choline as well as its downstream metabolites D9-betaine and D6-dimethyl glycine (D6-DMG) (Table 2A). By contrast, AUC was higher for D9-PC, D9-lyso-PC and D9-SPH after D9-POPC (Table 2B). Notably, this differentially applied to D9-PC sub-groups (Table 2C): whereas there were only minor differences for monounsaturated D9-C18:1-PC and no difference in D9-C22:6-PC, D9-POPC ingestion was followed by a major increase in omega-6 polyunsaturated D9-PC compounds (D9-C18:2-PC, D9-C20:4-PC) and in the omega-3 compound D9-C20:5-PC.

**Table 2:** Area under curve (AUC) of plasma D9-choline and its metabolites in response to water-soluble (D9-choline chloride, D9-GPC, D9-phosphocholine) and lipidic (D9-POPC) choline supplements

Supplement	D9-choline chloride	D9-GPC	D9-phospho-choline	D9-POPC
A: Water-soluble D9-choline metabolites				
D9-choline (0-6 h)	5.05 (4.52–6.04)	3.63 (3.11–5.00)	3.87 (3.12–5.90)	2.10 (1.84–2.43)***
D9-betaine (0-6d)	14.9 (13.1–16.4)	12.1 (11.6–15.4)	14.1(11.6–15.5)	4.75(3.56–6.16)***
D6-DMG (0-72 h)	2.13 (1.20–2.46)	1.80 (0.95–1.85)	1.49 (1.12–1.97)	0.32 (0.14–0.52)**
D9-TMAO (0-7d)	0.52 (0.37–1.61)	0.20 (0.05–0.43)	0.02 (0.00–0.04)	0.00 (0.00–0.16)^n.s^
B: D9-choline-labelled phospholipid classes				
D9-PC (0-7d)	1666 (1395–183)	1683 (1294–1898)	1623 (1451–1798)	2904 (2414–3071)***
D9-Lyso-PC (0-7d)	18.4 (14.8–25.0)	15.0 (10.5–16.1)	17.0 (15.9–17.5)	49.5 (37.8–57.8)***
D9-SPH (0-7d)	135 (103–147)	75 (42–94	119 (109–157)	164 (148–200)*
C: D9-PC molecular sub-classes				
D9-C18:1-PC (0-7d)	370 (286–443)	355 (271–380	372 (295–470	471 (364–527)*
D9-C18:2-PC (0-7d)	895 (789–940)	926 (708–1050)	841.16 (821–921)	1673 (1413–1909)***
D9-C20:4-PC (0-7d)	250 (216–325)	263 (229–348)	274 (229–319)	478 (468–628)***
D9-C20:5-PC (0-7d)	10.2 (7.4–18.0)	7.5 (6.7–11.8)	11.1 (8.0–20.7)	29.4 (22.3–40.8)*
D9-C22:6-PC (0-7d)	49.1 (41.7–53.2)	41.6 (35.0–45.3)	43.2 (38.7–56.6)	66.7 (50.0–74.2)^n.s^

Data are shown as medians (interquartile ranges) and are provided as µmol/l*h of plasma concentrations. For rapidly metabolised water-soluble compounds, shorter time intervals are indicated (0–72 h), compared to D9-labelled phospholipids (0–7d)

*AUC* area under the curve; *d* days; *D9-GPC* D9-glycerophosphocholine; *h* hours, *PC* phosphatidylcholine; *D9-POPC* D9-1-palmitoyl-1-oleoyl-PC; *D9-TMAO* D9-trimethylamine oxide; *n.s.* not significant

***p < 0.001; **p < 0.01*p < 0.01 vs water-soluble

### D3-PC from the PEMT pathway

To estimate the use of different D9-choline supplements for their contribution to methyl group metabolism and PC synthesis via PE methylation to form PC via the PEMT pathway, we investigated the plasma concentrations of D3-PC in response to D9-choline supplements. In this pathway, (D9-)betaine from (D9-)choline oxidation is required to synthesise (D3-) methionine, which after activation to (D3-)-S-adenosylmethionine is used to form D3- (and D6) PC [23]. As shown by supplementary Figure s5A, plasma concentrations of D3-PC correlated with the D9-enrichment of betaine as a methyl donor for this pathway, which was significant at 9 h. As shown in Figure s5B, appearance of D3-PC in plasma was uniformly delayed in response to all D9-choline supplements. In contrast to D9-PC, D3-PC did not show the rapid and maximal increase after D9-POPC administration. Moreover, there were no significant differences in the concentrations of D3-PC in response to the different D9-choline supplements.

## Discussion

Choline has been defined as an essential nutrient, with daily adequate intake (AI) values for healthy adult men of 550 mg according to NAM [15] and 400 mg according to EFSA [24]. Whilst choline is a critical nutrient throughout the population [25, 26], its adequate supply is particularly important in preterm infants with higher needs because of exponential growth, and in patients in whom choline deficiency is pathognomonic. The latter includes cystic fibrosis (CF) patients suffering from exocrine pancreas insufficiency and those with short bowl disease, both suffering from a disturbed enterohepatic cycle of bile PC, and the latter being additionally dependent on long-term total parenteral nutrition, still mostly devoid of free choline [4, 9]. For all these patients, optimal ways of choline supplementation need to be investigated. In this study, we administered four different deuterium-labelled choline (D9-choline) supplements, to follow their assimilation and the plasma kinetics of D9-choline and its deuterium-labelled metabolites. We compared free D9-choline in the form of its chloride salt, the water-soluble organic esters D9-glycerophosphocholine (D9-GPC) and D9-phosphocholine, whose native correlates predominate in milk [27], and 1-palmitoyl-2-oleoyl-D9-PC (D9-POPC) in analogy to the predominant PC compound in egg yolk.

All water-soluble supplements rapidly and similarly increased D9-choline and D9-betaine plasma levels, with maximum concentrations achieved at 0.5–1 h. This is consistent with the results on single administration of an AI dosage of unlabeled choline supplements in adults [28]. Consistently, (D9-)choline in the form of salts or water-soluble organic esters results in rapid absorption and via portal circulation and incomplete hepatic absorption increase in plasma D9-choline. In line with this process and the rapid plasma turnover of choline (~ 1 h), all these water-soluble choline compounds exhibited only a transient elevation of plasma choline concentrations after single oral intake. Consequently, a continuous elevation of plasma concentration to physiological values requires either a retarded choline formulation or a more frequent intake, i.e. during every meal, rather than once or twice per day [3, 28].

The increase in plasma D9-choline concentration after D9-POPC administration, however, was different in two ways. First, plasma concentrations increased later, peak concentrations were delayed from 0.5–1 h to ~ 1.5 h and the peaks were broader, indicating different mechanisms of D9-choline assimilation from lipidic D9-POPC. Such more retarded and longer lasting increase may be of benefit for achieving a balanced increase in plasma choline concentrations. After ingesting the AI value of choline (550 mg) in the form of egg-PC (4012 mg), maximum values of plasma choline were delayed to 3 h, but maximum concentrations were similar to water-soluble choline components. This discrepancy may be due to the higher dosage of ~ 7 mg/kg choline in our previous study, compared to 2.7 mg/kg D9-choline equivalent used here as a tracer, and the higher sensitivity of metabolic follow-up using deuterium-labelled compared to native compounds [3, 7, 28]. Such differences compared to water-soluble compounds are explained by the complex mechanisms involved: after formation of gastric chymus and participation of (D9-)PC in triglyceride (butter pretzel, Table s1) emulsification, all PC must first be cleaved, mainly by pancreatic phospholipase A2 (sPLA2IB), as only lyso-PC is absorbed by enterocytes. These further degrade a part of lyso-PC to release choline, but partly resynthesize PC from lyso-PC for chylomicron formation [29] (see below). Hence, D9-POPC was degraded to D9-lyso-PC within the duodenal lumen at the surface of lipid micelles, absorbed and further processed within enterocytes, finally resulting in a delayed and lower increase in plasma (D9-)choline. We used a standardised butter pretzel comprising ~ 12 g fat, content together with the ingestion of D9-choline components. Similar to higher choline dosages in the form of PC, higher triglyceride amounts may further delay the increase of free (D9-) choline in response to (D9-PO)PC.

Second, the maximum value and AUC of plasma D9-choline were lower after D9-POPC compared to water-soluble D9-choline components. It was suggested that lyso-PC is only partly degraded to free choline within the enterocytes, whereas ~ 50% are re-synthesised to PC, using available coenzyme A-activated fatty acids (acyl-CoA) and lyso-PC acyltransferases (LPCAT) [30]. From plasma analyses in humans, we cannot calculate D9-choline and D9-PC pools as is feasible in animal experiments [20]. Nevertheless, the lower AUC of D9-choline (-50%) and of its water-soluble downstream metabolites D9-betaine (-65%) and D6-DMG (-82%) together with a higher AUC of D9-PC (+ 50%) in response to D9-POPC compared to the water-soluble D9-choline compounds suggest that (D9-PO)PC administration partly circumvents full hydrolysis to D9-choline for PC synthesis de novo and the synthesis of downstream metabolites for methyl donation [31, 32]. Nevertheless, endogenous (D3-)PC and (D3-)choline synthesis via the PEMT pathway is not significantly inhibited in response to (D9-PO)PC as a choline supplement (see below).

### Plasma D9-PC and D3-PC metabolism in response to D9-choline supplements

PC is the major carrier for the plasma transport of long-chain polyunsaturated fatty acids (LC-PUFA), mainly arachidonic (ARA) and docosahexaenoic acid (DHA) to organs, via lipoproteins [33]. ARA and DHA deficiencies in preterm infants are associated with neonatal morbidity [34] and the clinical status of CF patients [10]. LC-PUFA are integrated in structural and functional membrane phospholipids of organs, like PC, phosphatidylethanolamine (PE) and others. Plasma transport of LC-PUFA via PC may, therefore, be essential for adequate development of the organs’ ‘lipidome’, especially that of the cerebrum, cerebellum and retina. Whereas hepatic very low-density lipoproteins (VLDL) comprise ~ 20% PC, chylomicrons contain ~ 8% PC as a coating so that their assembly within enterocytes requires PC formation. Such enterocytic PC synthesis partly originates from absorbed lyso-PC, using LPCAT3 that prefers polyunsaturated acyl-CoA as a substrate, and is, therefore, essential for plasma lipid transport of LC-PUFA [30]. Our results clearly show that LA, ARA, DHA and EPA are preferentially incorporated into (D9-)PC by the intestine, when (D9-PO)PC is used as a (D9-)choline supplement. In contrast, however, in the liver, DHA-PC is primarily and ARA-PC by 50% formed by methylation of PE via the PEMT pathway, requiring the formation of (D9-)betaine from (D9-)choline that is demethylated to (D6-) dimethylglycine for (D3-)methionine synthesis from homocysteine (Figs. 1B, 2A) [7].

In general, the formation of betaine, and its D9-enrichment correlated with the plasma concentrations of D3-PC (see supplementary Figure s5A). In this context, the lower increase in plasma D9-betaine and, subsequently, in D3-PC in response to D9-POPC, compared to its water-soluble D9-choline analogues, may be important for the homeostasis of methyl groups. In response to the ingestion of D9-POPC, compared to that of D9-choline chloride, D9-GPC and D9-phosphocholine, the generation of free D9-choline, D9-betaine and D6-dimethylglycine was decreased and, instead, (D9-PO)PC was used by the enterocytes via (D9-) lyso-PC re-acylation to PC for chylomicron formation. In line with this, D9-PC concentrations in plasma rapidly increase and maximal concentrations are highest after (D9-PO)PC as a choline supplement. Whilst D9-POPC resulted in lower plasma concentrations of free D9-choline, D9-betaine and D6-dimethylglycine (D6-DMG), it should be noticed that the administration of any water-soluble components not only normalises plasma choline concentrations, but may increase plasma betaine above physiological values [9, 35]. On the other hand, whereas the plasma concentrations of free (D9-)choline and (D9-)betaine were lower following (D9-PO)PC compared to water-soluble choline supplements, the synthesis of D3-PC via PE methylation by PEMT was not significantly decreased (Supplemental Fig. 5), suggesting that (D9-PO)PC as a choline source did not impair hepatic methyl group homeostasis.

### Differential kinetics of plasma D9-PC in response to D9-choline supplements

Administration of water-soluble D9-choline compounds resulted in an increase of plasma D9-PC that showed uniform peak concentrations at 24-33 h, and a plasma half-life of 2-3d as previously demonstrated after both enteral and parenteral D9-choline chloride administration [3, 7]. They all result in the preferential hepatic synthesis and secretion of D9-PC species that are dominated by linoleic (LA) (D9-C18:2-PC) and oleic acid (OA) (D9-C18:1-PC) PC species, followed by those containing an ARA (D9-C20:4-PC), DHA (D9-C22:6-PC) or eicosapentaenoic acid (EPA) residue (D9-C20:5-PC) as described before [3, 7, 10].

Such fatty acid specificity is consistent with the PC synthesis de novo by adult liver, and contrasts to the preferential synthesis of C22:6-PC and C20:4-PC synthesis by the PEMT pathway using betaine as a methyl donor [7]. However, such fatty acid specificity does not apply to other organs, foetuses and newborns, where large amounts of C22:6-PC and C20:4-PC are derived from de novo PC synthesis, depending on fatty acid availability [1, 20, 35].

Importantly, assimilation of D9-POPC resulted in an earlier (9 h) and twofold higher peak concentration of D9-PC without plateau and an increased AUC. This indicates that (D9-)choline assimilation from (D9-PO)PC is faster with respect to plasma (D9-)PC increase, suggesting preferential use of ingested (D9-PO)PC for the (D9-)PC moiety of chylomicrons (see above). The similarly rapid decrease of D9-PC after D9-POPC and other compounds, reaching similarly low levels at 72 h (see Fig. 3a, insert), suggests that (D9-PO)PC results in higher (D9-) PC disposal in other organs. This is consistent with animal experiments showing high accretion of plasma PC by the brain and lung [20]. Finally, our data suggest that the intestine does not have a reservoir function for choline or PC like the liver, but only a ‘permissive’ function for peripheral supply derived from actually administered choline sources.

### Impact of different choline supplements on endogenous PC synthesis by PEMT

Our data show that, in response to (D9-PO)PC, the plasma concentrations of free (D9-)choline and (D9-)betaine are decreased but their hepatic use for the synthesis of (D3-)methionine as a methyl donor and (D3-)PC synthesis from PE via the PEMT pathway is not decreased. Hence, hepatic secretion of VLDL, where both PC synthesis de novo and via the PEMT pathway are essential [23], should not be impaired by POPC supplementation compared to supplementation with water-soluble choline compounds. The contribution of the PEMT pathway to PC synthesis is low in many humans, just like in adult males studied here, e.g. in postmenopausal women, women with characteristic single nucleotide polymorphisms (SNPs) and, particularly, preterm infants. Notably, such individuals with low PEMT (e.g. SNP rs12325817) activity only suffer from decreased VLDL formation and hepatosteatosis in severe choline deficiency [36]. Moreover, the selectivity of ARA- and DHA-PC synthesis via the PEMT pathway only exists in the liver, whereas foetal (and preterm infant) requirements and plasma concentrations of ARA- and DHA-PC are high in spite low PEMT activity, and extrahepatic synthesis of these PC components is high, although they do not express PEMT (lung, intestine) [20, 29]. Our data are consistent with this, as after D9-POPC, there was a rapid and increased formation of polyunsaturated D9-PC, with increased contents not only of LA, but also of ARA, DHA and EPA.

### Potential consequences of choline supplementation via PC instead of water-soluble components

The assimilation of D9-POPC, via cleavage to D9-lyso-PC, preferential small intestinal re-acylation to D9-PC and chylomicron assembly (see above), contrasted the hepatic D9-choline, -betaine, -PC and -VLDL metabolism as represented by the other D9-choline supplements. Although D9-POPC belongs to the D9-C18:1-PC subgroup, D9-C18:1-PC concentration was not increased here, but contrary to the other D9-choline supplements, remained a minor fraction of plasma D9-PC (see Figs. 4A and 5A). Instead, D9-POPC resulted in twofold to –threefold increases in D9-PC comprising LA, ARA and EPA. As the butter pretzels’ fat was dominated by OA (24%) over LA (22%), ARA (0.1%) and DHA (0%), the domination of polyunsaturated D9-PC is explained by the molecular specificity of LPCAT3 [31]. Such specificity of LPCAT3 suggests that, simultaneous supplementation of POPC as well as ARA and DHA at the expense of LA, might increase the ARA and DHA supply of organs. Further investigation is required to assess the quantitative impact of such combinations of increased LC-PUFA and reduced LA in combination with PC as a choline supplement.

### Formation of (D9-)TMAO in response to (D9-)choline supplements

In addition to the efficacy of a choline supplement, safety is paramount. Therefore, we also investigated the concentration of D9-TMAO, the hepatic oxidation product of D9-TMA that is a bacterial D9-choline degradation product. TMAO has been described as a cardiovascular risk factor [17]. Notably, neither D9-POPC nor D9-phosphocholine resulted in detectable D9-TMAO concentrations. Whereas this was previously shown for (egg-)PC [28], absence of TMAO formation from phosphocholine is a new finding. Notably, D9-phosphocholine showed the earliest peak concentration of plasma D9-choline (0.75 h), compared to any other choline compound. Further research is required here to address phosphocholine assimilation as a choline supplement, possibly occurring earlier and more proximally in the small intestine than other compounds.

Differences in absorption may also apply to (D9-) choline chloride and (D9-)GPC, where the latter showed a later increase of TMAO (> 6 h) that was not detected in a previous study at 0–6 h [28]. Unfortunately, phosphocholine as a rapidly absorbed choline compound is not commercially available in nutritional bulk quantities, but taking the effects on (D9-)choline, (D9-)betaine and (D9-)PC levels as well as those on D9-PC fatty acid kinetics and (D9-) TMAO formation into account, we conclude that (PO)PC might be the best suitable supplement for adults, possibly in combination with phosphocholine. A water-soluble choline supplement in combination with PC appears important, as adults with choline deficiency also have low betaine concentrations in plasma and free choline is an important source of betaine as the major methyl donor [1, 3, 6, 20].

### Limitations

The main limitation of the study is the small number of participants which limited the power to detect smaller differences in plasma kinetics, particularly between the different water-soluble supplements. Additionally, we only assessed healthy male adults, excluding the oestrogen impact on choline metabolism via higher PEMT expression in pre-menopausal women [36]. Moreover, adult men’s metabolic rate does not represent that of the fast growing preterm infant, nor is the impact of exocrine pancreas dysfunction, small intestinal pathologies and dysbiosis represented by these volunteers [9]. Nevertheless, a significant number of healthy pre-menopausal women have deleterious single nucleotide polymorphisms resulting in low PEMT expression like males [36]. Finally, in spite of exocrine pancreas insufficiency in CF patients, and massive fecal choline losses due to pancreatic phospholipase A2 deficiency, these patients profit from choline administration in the form of PC as intestinal phospholipase activity is not fully absent [9, 37, 38]. Hence, whilst further studies must follow with specific clinical cohorts, our data provide a frame work to optimise choline supplementation (Table 3).

**Table 3:** Time to peak (h) of concentrations of D9-choline and D9-choline metabolites in response to different D9-choline tracers

Plasma compound	Supplement			
	D9-choline chloride	D9-GPC	D9-phosphoryl-choline	D9-POPC
D9-Choline	1.0 (0.5–1.13)	1.0 (0.9–1.1)	0.8 (0.5–1.0)	1.5 (1.4–2.3)
D9-Betaine	1.5 (1.0–2.5)	1.5 (1.4–1.6)	1.5 (1.4–2.0)	3.5 (2.0–5.3)
D9-PC	28.5 (24.0–33.0)	28.5 (24.0–33.0)	24.0 (24.0–33.0)	9.0 (9.0–24.0)
D9-TMAO	1.3 (1.0–5.3)	9.0 (5.5–9.0)	5.0 (4.5.-19.5)	9.0 (6.0–9.0)^a^

Data are shown as medians and interquartile ranges

*D9-GPC* D9-glycerophosphorylcholine; *h* hours; *D9-POPC* D9-1-palmitoyl-2-oleoyl-phosphatidylcholine, *D9-PC* D9-phosphatidylcholine; *D9-TMAO* D9-trimethylamine oxide

^a^Based on a limited number of samples with detectable D9-TMAO

## Conclusion

There was a retarded increase in plasma D9-choline and D9-betaine as well as higher peak concentrations and AUCs of D9-PC and virtually absent D9-TMAO formation after D9-POPC and D9-phosphocholine administration. The use of (D9-PO)PC as a choline supplement for preferential enterocytic synthesis of polyunsaturated (D9-)PC implies the option of optimising DHA, and ARA supply for preterm infants [1, 22], by co-supplementation with lipidic (PC) rather than water-soluble choline supplements. Therefore, PC—alone or in combination—may be a suitable choline supplement for adults, and potentially other patient groups. All data are included in this article or the supplementary information.

## Supplementary Information

Below is the link to the electronic supplementary material.

Supplementary file1 (PPTX 195 KB)

## Abbreviations

AI: Adequate intake
CF: Cystic fibrosis
EDTA: Ethylenediaminetetraacetate
EFSA: European Food Safety Authority
FDA: US-Food and Drug Administration
αGPC: Alpha-glycerophosphocholine
GRAS: Generally recognised as safe
NAM: National Academy of Medicine of the USA
PC: Phosphatidylcholine
(D9-)POPC: (D9-)1-palmitoyl-2-oleoyl-PC
(D9-)C18:1-PC: (D9-choline labelled)PC containing an oleic acid residue
(D9-)C18:2-PC: (D9-choline labelled)PC containing a linoleic acid residue
(D9-)C20:4-PC: (D9-choline labelled)PC containing an arachidonic acid residue
(D9-)C20:5PC: (D9-choline labelled)PC containing an eicosapentaenoic acid residue
(D9-)C22:6PC: (D9-choline labelled)PC containing a docosahexaenoic acid residue
D9-SPH: D9-labelled sphingomyelin
D9-TMAO: D9-labelled trimethylamine oxide

## References

[1] Bernhard W, Poets CF, Franz AR (2019). Choline and choline-related nutrients in regular and preterm infant growth. Eur J Nutr.

[2] Wiedeman AM, Barr SI, Green TJ, Xu Z, Innis SM, Kitts DD (2018). Dietary choline intake: current state of knowledge across the life cycle. Nutrients.

[3] Bernhard W, Lange R, Graepler-Mainka U, Engel C, Machann J, Hund V, Shunova A, Hector A, Riethmüller J (2019). Choline supplementation in cystic fibrosis-the metabolic and clinical impact. Nutrients.

[4] Zeisel SH (2006). Choline: critical role during fetal development and dietary requirements in adults. Annu Rev Nutr.

[5] Oshida K, Shimizu T, Takase M, Tamura Y, Shimizu T, Yamashiro Y (2003). Effects of dietary sphingomyelin on central nervous system myelination in developing rats. Pediatr Res.

[6] Innis SM, Davidson AG, Bay BN, Slack PJ, Hasman D (2011). Plasma choline depletion is associated with decreased peripheral blood leukocyte acetylcholine in children with cystic fibrosis. Am J Clin Nutr.

[7] Pynn CJ, Henderson NG, Clark H, Koster G, Bernhard W, Postle AD (2011). Specificity and rate of human and mouse liver and plasma phosphatidylcholine synthesis analyzed in vivo. J Lipid Res.

[8] Ozarda Ilcol Y, Uncu G, Ulus IH (2002). Free and phospholipid-bound choline concentrations in serum during pregnancy, after delivery and in newborns. Arch Physiol Biochem.

[9] Bernhard W (2020). Choline in cystic fibrosis: relations to pancreas insufficiency, enterohepatic cycle, PEMT and intestinal microbiota. Eur J Nutr.

[10] Grothe J, Riethmuller J, Tschurtz SM, Raith M, Pynn CJ, Stoll D, Bernhard W (2015). Plasma phosphatidylcholine alterations in cystic fibrosis patients: impaired metabolism and correlation with lung function and inflammation. Cell Physiol Biochem.

[11] Li Z, Agellon LB, Vance DE (2005). Phosphatidylcholine homeostasis and liver failure. J Biol Chem.

[12] Bernhard W, Shunova A, Machann J, Grimmel M, Haack TB, Utz P, Graepler-Mainka U (2021). Resolution of severe hepatosteatosis in a cystic fibrosis patient with multifactorial choline deficiency: a case report. Nutrition.

[13] Institute of Medicine Standing Committee on the Scientific Evaluation of Dietary Reference I, its Panel on Folate OBV, Choline (1998). The National Academies Collection: Reports funded by National Institutes of Health. Dietary reference intakes for thiamin, riboflavin, niacin, vitamin B(6), folate, vitamin B(12), pantothenic acid, biotin, and choline.

[14] Zeisel SH, da Costa KA (2009). Choline: an essential nutrient for public health. Nutr Rev.

[15] Institute of Medicine (US) (1998) Standing committee on the scientific evaluation of dietary reference intakes and its panel on folate, other B vitamins, and choline. Dietary Reference Intakes for Thiamin, Riboflavin, Niacin, Vitamin B6, Folate, Vitamin B12, Pantothenic Acid, Biotin, and Choline. Washington (DC): National Academies Press (US) 23193625

[16] da Costa KA, Corbin KD, Niculescu MD, Galanko JA, Zeisel SH (2014). Identification of new genetic polymorphisms that alter the dietary requirement for choline and vary in their distribution across ethnic and racial groups. Faseb j.

[17] Wang Z, Klipfell E, Bennett BJ, Koeth R, Levison BS, Dugar B, Feldstein AE, Britt EB, Fu X, Chung YM, Wu Y, Schauer P, Smith JD, Allayee H, Tang WH, DiDonato JA, Lusis AJ, Hazen SL (2011). Gut flora metabolism of phosphatidylcholine promotes cardiovascular disease. Nature.

[18] Tang WH, Wang Z, Levison BS, Koeth RA, Britt EB, Fu X, Wu Y, Hazen SL (2013). Intestinal microbial metabolism of phosphatidylcholine and cardiovascular risk. N Engl J Med.

[19] Dombrowsky H, Clark GT, Rau GA, Bernhard W, Postle AD (2003). Molecular species compositions of lung and pancreas phospholipids in the cftr(tm1HGU/tm1HGU) cystic fibrosis mouse. Pediatr Res.

[20] Bernhard W, Raith M, Shunova A, Lorenz S, Böckmann K, Minarski M, Poets CF, Franz AR (2022). Choline kinetics in neonatal liver, brain and lung-lessons from a rodent model for neonatal care. Nutrients.

[21] Cheng WL, Holmes-McNary MQ, Mar MH, Lien EL, Zeisel SH (1996). Bioavailability of choline and choline esters from milk in rat pups. J Nutr Biochem.

[22] Böckmann KA, von Stumpff A, Bernhard W, Shunova A, Minarski M, Frische B, Warmann S, Schleicher E, Poets CF, Franz AR (2021). Fatty acid composition of adipose tissue at term indicates deficiency of arachidonic and docosahexaenoic acid and excessive linoleic acid supply in preterm infants. Eur J Nutr.

[23] Vance DE (2013). Physiological roles of phosphatidylethanolamine N-methyltransferase. Biochim Biophys Acta.

[24] EFSA Panel on Dietetic Products N Allergies (2016). Dietary reference values for choline. EFSA J.

[25] Resseguie ME, da Costa KA, Galanko JA, Patel M, Davis IJ, Zeisel SH (2011). Aberrant estrogen regulation of PEMT results in choline deficiency-associated liver dysfunction. J Biol Chem.

[26] Guerrerio AL, Colvin RM, Schwartz AK, Molleston JP, Murray KF, Diehl A, Mohan P, Schwimmer JB, Lavine JE, Torbenson MS, Scheimann AO (2012). Choline intake in a large cohort of patients with nonalcoholic fatty liver disease. Am J Clin Nutr.

[27] Maas C, Franz AR, Shunova A, Mathes M, Bleeker C, Poets CF, Schleicher E, Bernhard WJEJON (2017). Choline and polyunsaturated fatty acids in preterm infants’ maternal milk. Eur J Nutr.

[28] Böckmann KA, Franz AR, Minarski M, Shunova A, Maiwald CA, Schwarz J, Gross M, Poets CF, Bernhard W (2022). Differential metabolism of choline supplements in adult volunteers. Eur J Nutr.

[29] Nilsson Å, Duan RD (2019). Pancreatic and mucosal enzymes in choline phospholipid digestion. Am J Physiol Gastrointest Liver Physiol.

[30] Shi C, Qiao S, Wang S, Wu T, Ji G (2019). Recent progress of lysophosphatidylcholine acyltransferases in metabolic disease and cancer. Int J Clin Exp Med.

[31] Zeisel SH (1981). Dietary choline: biochemistry, physiology, and pharmacology. Annu Rev Nutr.

[32] Hollenbeck CB (2012). An introduction to the nutrition and metabolism of choline. Cent Nerv Syst Agents Med Chem.

[33] Bernhard W, Maas C, Shunova A, Mathes M, Bockmann K, Bleeker C, Vek J, Poets CF, Schleicher E, Franz AR (2018). Transport of long-chain polyunsaturated fatty acids in preterm infant plasma is dominated by phosphatidylcholine. Eur J Nutr.

[34] Martin CR, Dasilva DA, Cluette-Brown JE, Dimonda C, Hamill A, Bhutta AQ, Coronel E, Wilschanski M, Stephens AJ, Driscoll DF, Bistrian BR, Ware JH, Zaman MM, Freedman SD (2011). Decreased postnatal docosahexaenoic and arachidonic acid blood levels in premature infants are associated with neonatal morbidities. J Pediatr.

[35] Bernhard W, Bockmann K, Maas C, Mathes M, Hovelmann J, Shunova A, Hund V, Schleicher E, Poets CF, Franz AR (2019). Combined choline and DHA supplementation: a randomized controlled trial. Eur J Nutr.

[36] Fischer LM, da Costa KA, Kwock L, Galanko J, Zeisel SH (2010). Dietary choline requirements of women: effects of estrogen and genetic variation. Am J Clin Nutr.

[37] Innis SM, Davidson AG, Melynk S, James SJ (2007). Choline-related supplements improve abnormal plasma methionine-homocysteine metabolites and glutathione status in children with cystic fibrosis. Am J Clin Nutr.

[38] Chen AH, Innis SM, Davidson AG, James SJ (2005). Phosphatidylcholine and lysophosphatidylcholine excretion is increased in children with cystic fibrosis and is associated with plasma homocysteine, S-adenosylhomocysteine, and S-adenosylmethionine. Am J Clin Nutr.

