# Supplementary material for: Different choline supplement metabolism in adults using deuterium labelling
Source: Eur J Nutr. 2023 Feb 25;62(4):1795–807. doi: 10.1007/s00394-023-03121-z (PMC10195734; doi:10.1007/s00394-023-03121-z)
Supplement: Supplementary file 1 — Supplementary file1 (PPTX 195 KB) [file 394_2023_3121_MOESM1_ESM.pptx]

## Slide 1
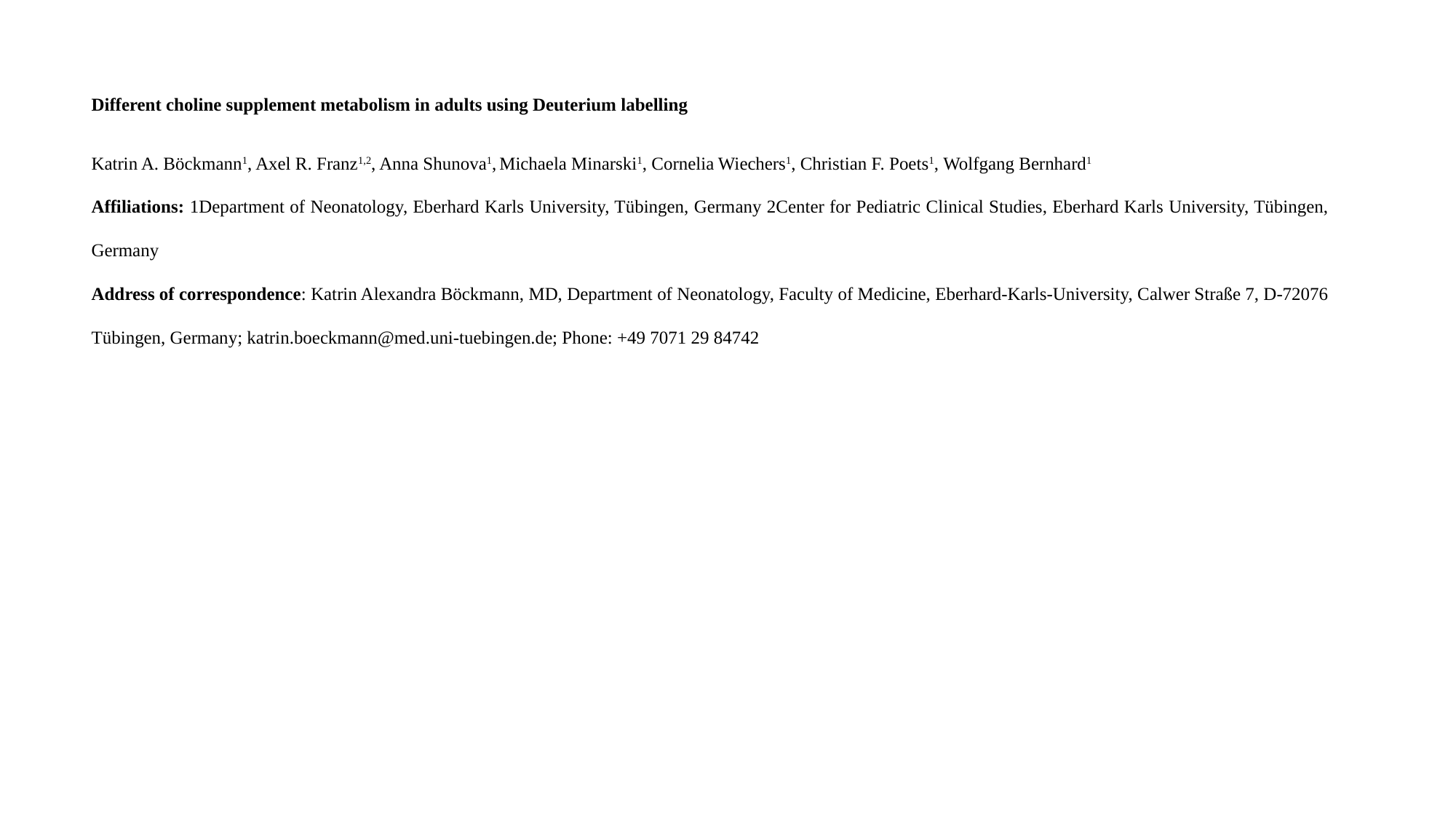

Different choline supplement metabolism in adults using Deuterium labelling
Katrin A. Böckmann1, Axel R. Franz1,2, Anna Shunova1, Michaela Minarski1, Cornelia Wiechers1, Christian F. Poets1, Wolfgang Bernhard1
Affiliations: 1Department of Neonatology, Eberhard Karls University, Tübingen, Germany 2Center for Pediatric Clinical Studies, Eberhard Karls University, Tübingen, Germany
Address of correspondence: Katrin Alexandra Böckmann, MD, Department of Neonatology, Faculty of Medicine, Eberhard-Karls-University, Calwer Straße 7, D-72076 Tübingen, Germany; katrin.boeckmann@med.uni-tuebingen.de; Phone: +49 7071 29 84742

## Slide 2
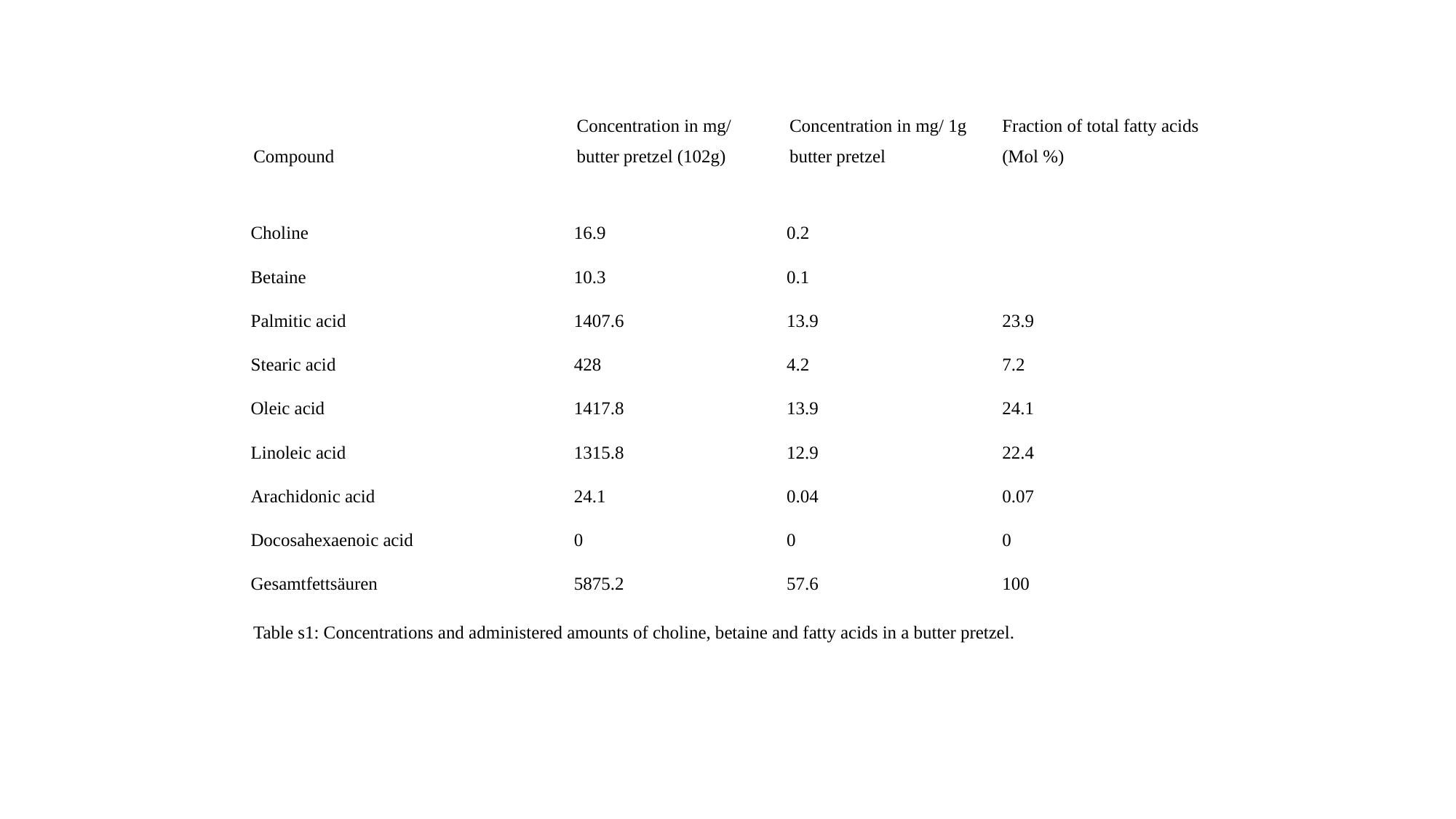

| Compound | Concentration in mg/ butter pretzel (102g) | Concentration in mg/ 1g butter pretzel | Fraction of total fatty acids (Mol %) |
| --- | --- | --- | --- |
| Choline | 16.9 | 0.2 | |
| Betaine | 10.3 | 0.1 | |
| Palmitic acid | 1407.6 | 13.9 | 23.9 |
| Stearic acid | 428 | 4.2 | 7.2 |
| Oleic acid | 1417.8 | 13.9 | 24.1 |
| Linoleic acid | 1315.8 | 12.9 | 22.4 |
| Arachidonic acid | 24.1 | 0.04 | 0.07 |
| Docosahexaenoic acid | 0 | 0 | 0 |
| Gesamtfettsäuren | 5875.2 | 57.6 | 100 |
Table s1: Concentrations and administered amounts of choline, betaine and fatty acids in a butter pretzel.

## Slide 3
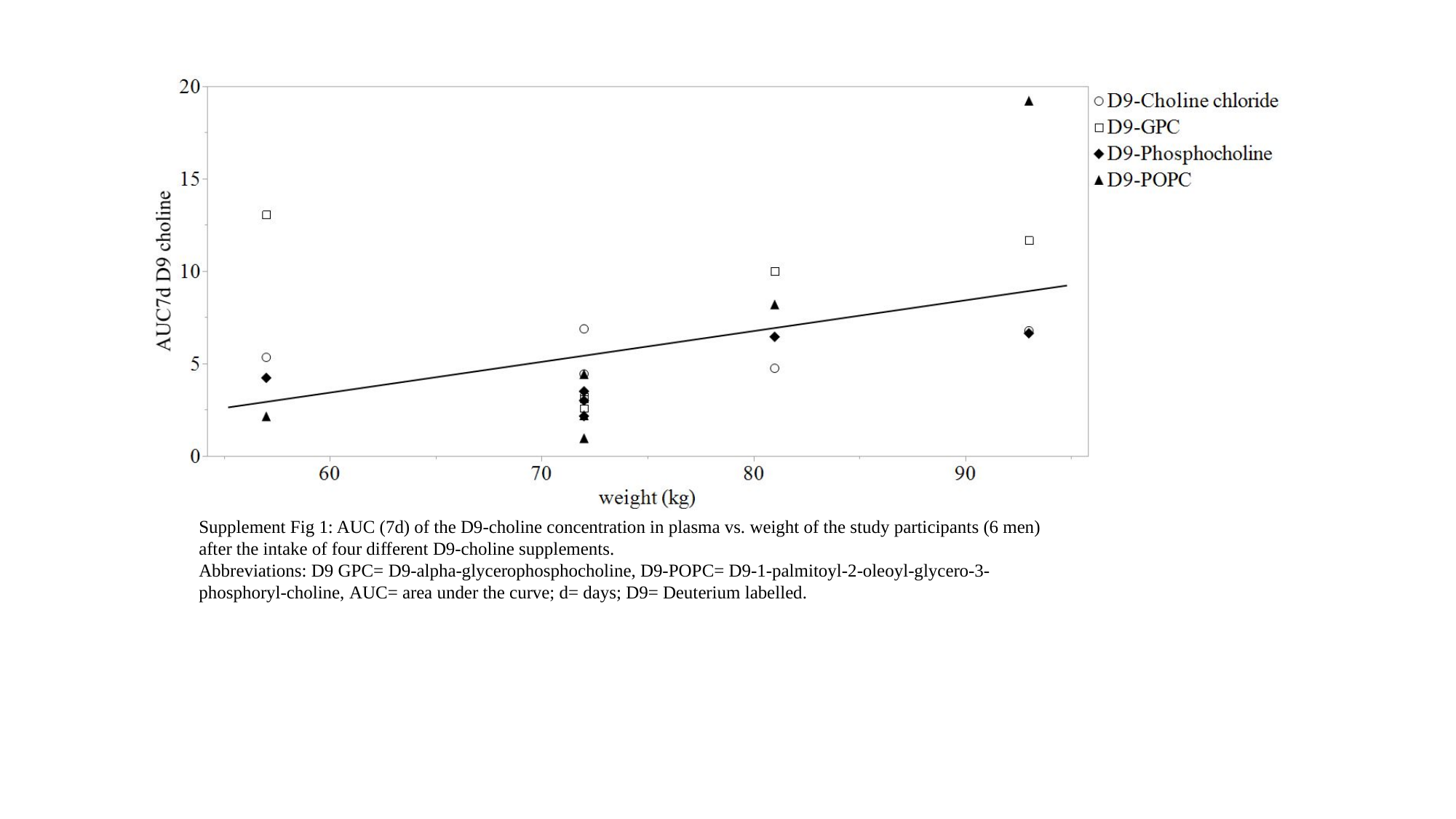

Supplement Fig 1: AUC (7d) of the D9-choline concentration in plasma vs. weight of the study participants (6 men) after the intake of four different D9-choline supplements.
Abbreviations: D9 GPC= D9-alpha-glycerophosphocholine, D9-POPC= D9-1-palmitoyl-2-oleoyl-glycero-3-phosphoryl-choline, AUC= area under the curve; d= days; D9= Deuterium labelled.

## Slide 4
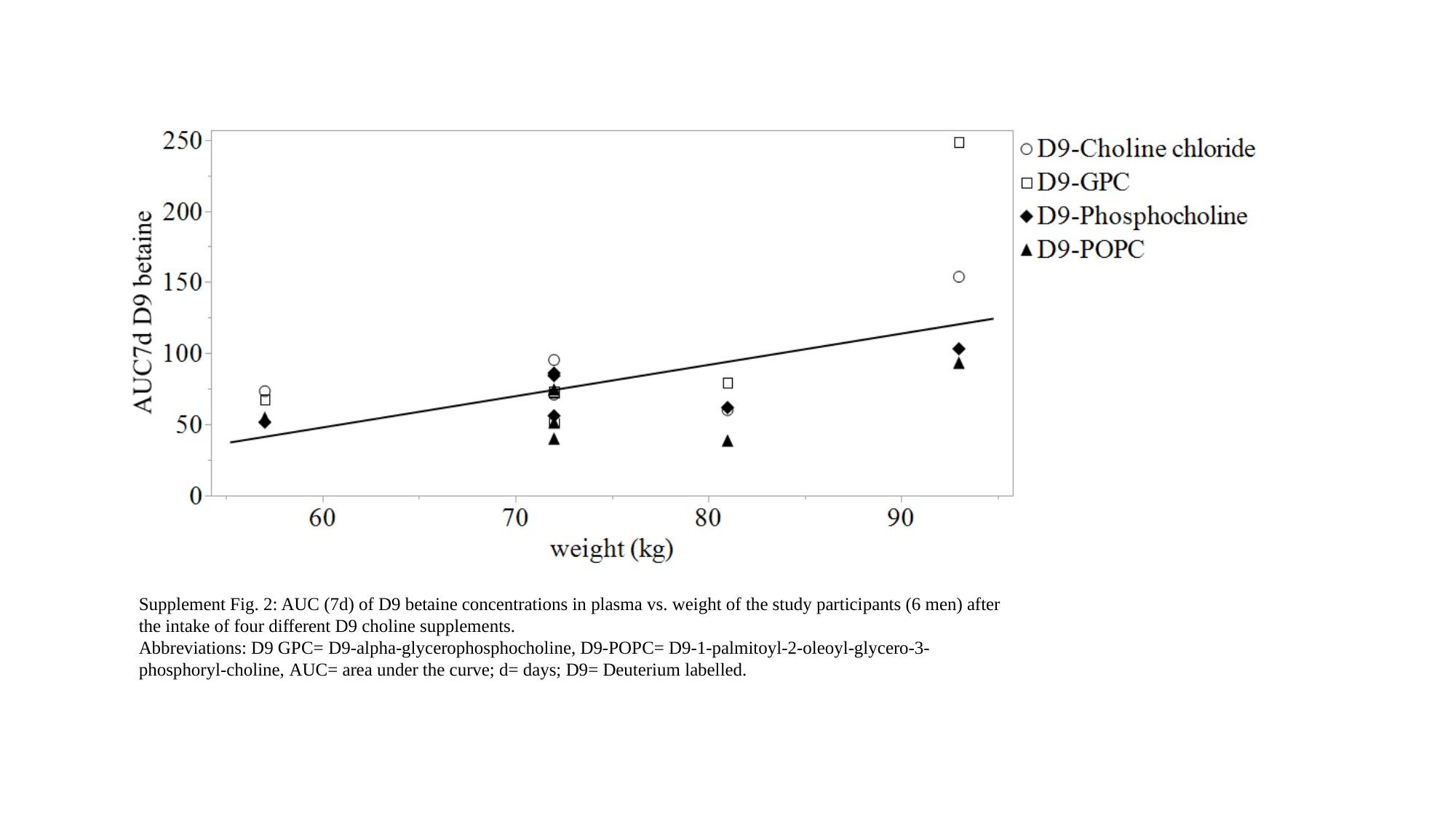

Supplement Fig. 2: AUC (7d) of D9 betaine concentrations in plasma vs. weight of the study participants (6 men) after the intake of four different D9 choline supplements.
Abbreviations: D9 GPC= D9-alpha-glycerophosphocholine, D9-POPC= D9-1-palmitoyl-2-oleoyl-glycero-3-phosphoryl-choline, AUC= area under the curve; d= days; D9= Deuterium labelled.

## Slide 5
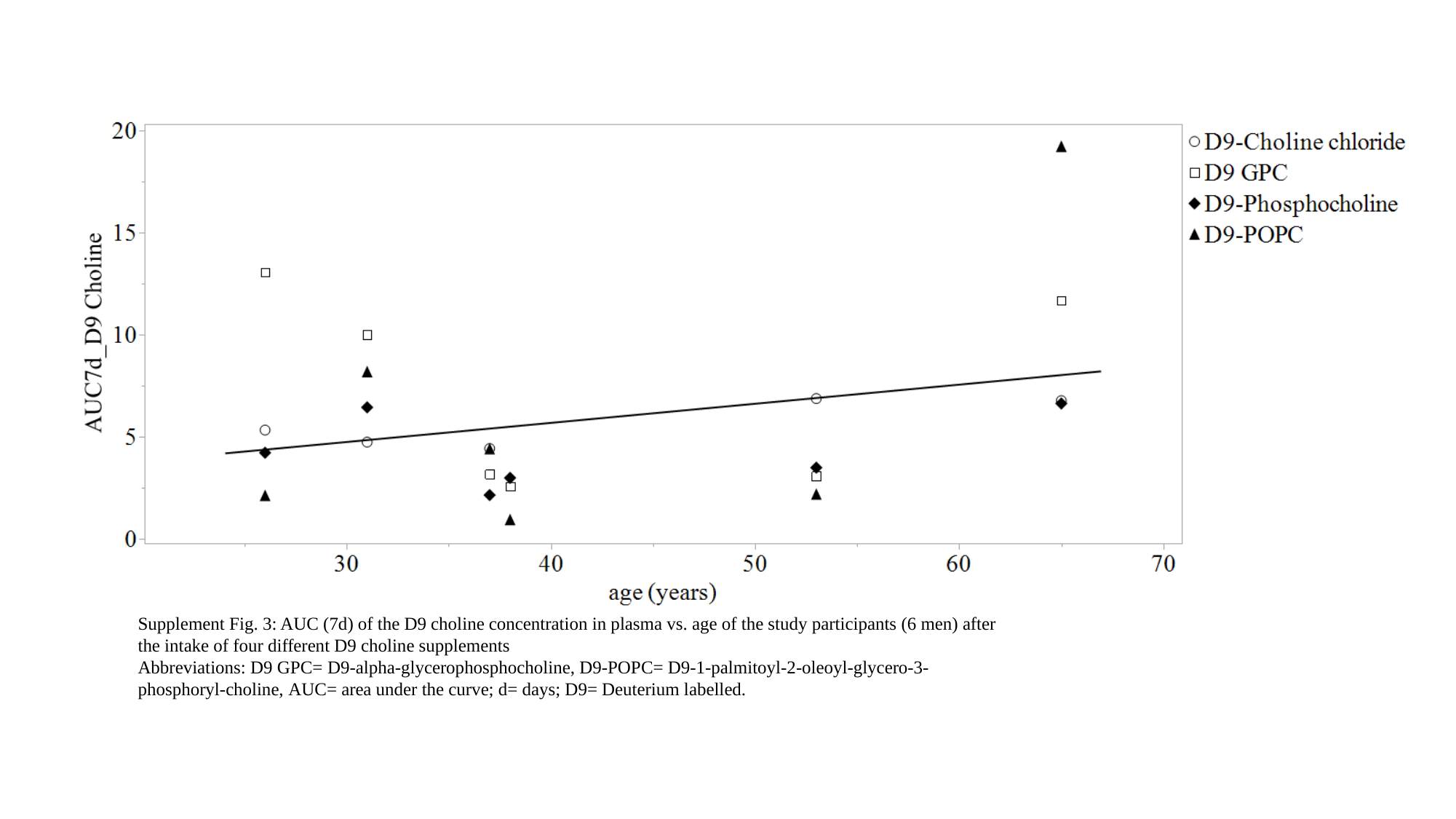

Supplement Fig. 3: AUC (7d) of the D9 choline concentration in plasma vs. age of the study participants (6 men) after the intake of four different D9 choline supplements
Abbreviations: D9 GPC= D9-alpha-glycerophosphocholine, D9-POPC= D9-1-palmitoyl-2-oleoyl-glycero-3-phosphoryl-choline, AUC= area under the curve; d= days; D9= Deuterium labelled.

## Slide 6
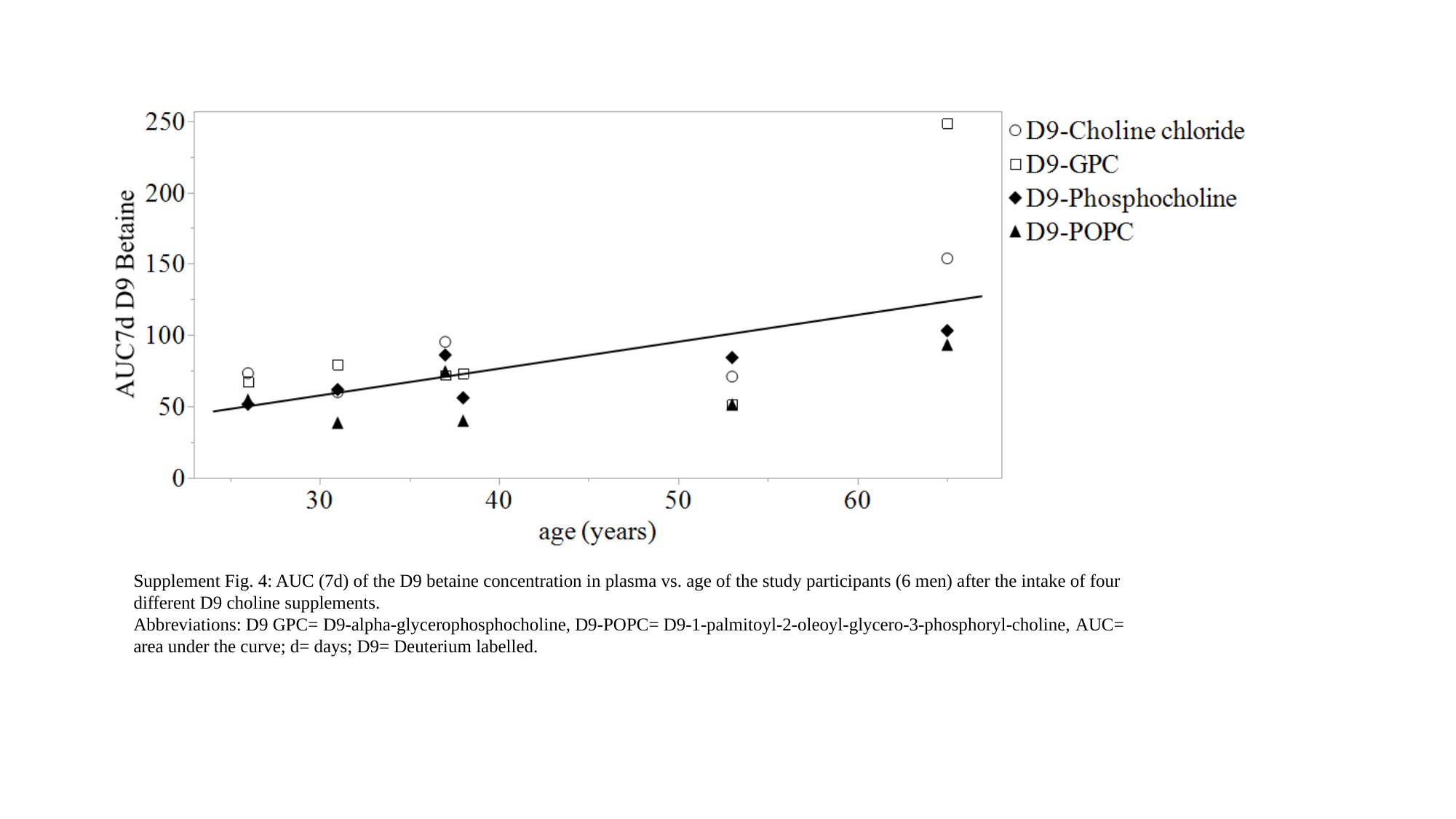

Supplement Fig. 4: AUC (7d) of the D9 betaine concentration in plasma vs. age of the study participants (6 men) after the intake of four different D9 choline supplements.
Abbreviations: D9 GPC= D9-alpha-glycerophosphocholine, D9-POPC= D9-1-palmitoyl-2-oleoyl-glycero-3-phosphoryl-choline, AUC= area under the curve; d= days; D9= Deuterium labelled.

## Slide 7
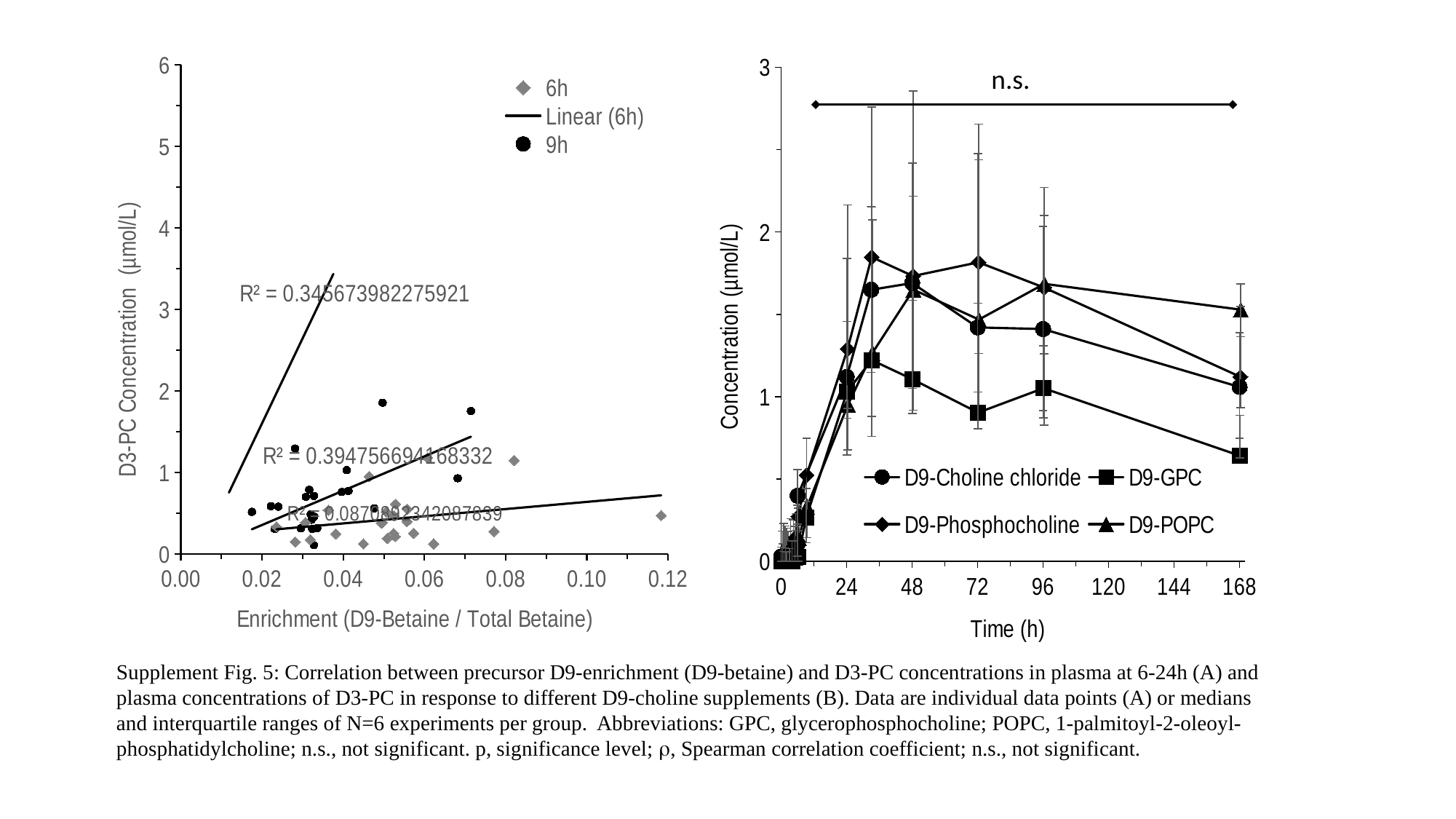

### Chart
| Category | D9-Choline chloride | D9-GPC | D9-Phosphocholine | D9-POPC |
|---|---|---|---|---|
### Chart
| Category | | | |
|---|---|---|---|n.s.
Supplement Fig. 5: Correlation between precursor D9-enrichment (D9-betaine) and D3-PC concentrations in plasma at 6-24h (A) and plasma concentrations of D3-PC in response to different D9-choline supplements (B). Data are individual data points (A) or medians and interquartile ranges of N=6 experiments per group. Abbreviations: GPC, glycerophosphocholine; POPC, 1-palmitoyl-2-oleoyl-phosphatidylcholine; n.s., not significant. p, significance level; r, Spearman correlation coefficient; n.s., not significant.
